# Usefulness of manufactured tomato extracts in the diagnosis of tomato sensitization: Comparison with the prick-prick method

**DOI:** 10.1186/1476-7961-6-1

**Published:** 2008-01-09

**Authors:** Ángel Ferrer, Ángel J Huertas, Carlos H Larramendi, Jose L García-Abujeta, Joan Bartra, Jose R Lavín, Carmen Andreu, Juan A Pagán, María A López-Matas, Enrique Fernández-Caldas, Jerónimo Carnés

**Affiliations:** 1 Allergy Service, Hospital Vega Baja, Orihuela, Alicante, Spain; 2 Allergy Service, Hospital General Básico de la Defensa, Cartagena, Murcia, Spain; 3 Allergy Service, Hospital Marina Baixa, Villajoyosa, Alicante, Spain; 4 Allergy Service, Hospital Clinic de Barcelona, Barcelona, Spain; 5 Allergy Service, Hospital General Básico de la Defensa, Valencia, Spain; 6 Allergy Service, Hospital Virgen de la Arrixaca, Murcia, Spain; 7 Research & Development Department, Laboratorios LETI, S.L., Tres Cantos, Madrid, Spain

## Abstract

**Background:**

Commercial available skin prick test with fruits can be negative in sensitized or allergic patients due to a reduction in biological activity during the manufacturing process. Prick-prick tests with fresh foods are often preferred, but they are a non-standardized procedure. The usefulness of freeze-dried extracts of Canary Islands tomatoes, comparing the wheal sizes induced by prick test with the prick-prick method in the diagnosis of tomato sensitization has been analyzed.

The objective of the study was to assess the potential diagnostic of freeze-dried extracts of Canary Islands tomatoes, comparing the wheal sizes induced by prick test with the prick-prick method.

**Methods:**

Two groups of patients were analyzed: Group I: 26 individuals reporting clinical symptoms induced by tomato contact or ingestion. Group II: 71 control individuals with no symptoms induced by tomato: 12 of them were previously skin prick test positive to a tomato extract, 39 were atopic and 20 were non-atopic. All individuals underwent prick-prick with fresh ripe peel Canary tomatoes and skin prick tested with freeze-dried peel and pulp extracts obtained from peel and pulp of Canary tomatoes at 10 mg/ml. Wheal sizes and prick test positivity (≥ 7 mm^2^) were compared between groups.

**Results:**

In group I, 21 (81%) out of 26 patients were prick-prick positive. Twenty patients (77%) had positive skin prick test to peel extracts and 12 (46%) to pulp extracts. Prick-prick induced a mean wheal size of 43.81 ± 40.19 mm^2 ^compared with 44.25 ± 36.68 mm^2 ^induced by the peel extract (Not significant), and 17.79 ± 9.39 mm^2 ^induced by the pulp extract (p < 0.01).

In group II, 13 (18%) out of 71 control patients were prick-prick positive. Twelve patients (all of them previously positive to peel extract) had positive skin prick test to peel and 3 to pulp. Prick-prick induced a mean wheal size of 28.88 ± 13.12 mm^2 ^compared with 33.17 ± 17.55 mm^2 ^induced by peel extract (Not significant), and 13.33 ± 4.80 mm^2 ^induced by pulp extract (p < 0.05 with peel extract and prick-prick).

**Conclusion:**

Canary peel tomato extract seems to be as efficient as prick-prick tests with ripe tomatoes to diagnose patients sensitized to tomato. The wheal sizes induced by prick-prick and peel extracts were very similar and showed a high correlation coefficient.

## Background

Two different methods are usually used for *in vivo *diagnosis of food allergy, skin prick tests (SPT) and food challenges. SPT offers several advantages: they are easier to perform and are widely available, but positive results only indicate the existence of food sensitization and not food allergy. Although allergy can only be confirmed by food challenges, detection of sensitization is an essential clue for the study of allergy [1]. The identification of all food sensitized individuals is essential to understand the exact meaning of sensitization and to establish its relationship with clinical allergy. Therefore the necessity to improve the available diagnostic tools does not need to be emphasized [2]. Currently, SPT can be performed either with natural fresh foods (prick-prick) or with manufactured extracts. Available commercial food extracts can be negative in sensitized or allergic patients due to a reduction in biological activity during the manufacturing process, mainly during homogenization, extraction and filtration processes.) [3,4] SPT with fresh foods (prick-prick tests) are considered the gold standard to assess sensitization [5]. The use of fresh foods is a non-standardized process, the availability of the source material is irregular and differences in their allergenic composition within the same food depending on the variety, state of ripeness, conservation process and cooking method among other factors may exist [6].

In the last years, some studies have reported that several carefully prepared manufactured food extracts induced skin reactions similar to prick-prick tests.) [3,4,7], but results may vary depending on the studied population [8]. It has been demonstrated that tomato sensitization is frequent in Spain [9], but the efficiency of commercial tomato extracts compared with prick-prick fresh tomatoes remains unknown.

The objective of the study was to compare the wheal sizes induced by prick tests containing freeze-dried extracts of Canary Islands tomatoes against prick-prick using fresh tomatoes in a population reporting symptoms with tomato.

## Methods

### Patient population

A total of 97 subjects who accepted to participate in the study, were recruited in the Allergy Units from the following centers: Hospital Clínic de Barcelona, Hospital General Básico de la Defensa, Valencia, Hospital Marina Baixa, Villajoyosa, Hospital de la Vega Baja, Orihuela and Hospital General Básico de la Defensa, Cartagena; all located along the Mediterranean Coast of Spain. All participant centers intended to include at least 5 subjects reporting symptoms with tomato and between 5 and 15 control individuals. All subjects were recruited and tested within a week to provide uniform conditions of testing with fresh foods. The study was conducted with the approval of the Hospital de la Vega Baja (Orihuela, Alicante) ethics committee.

Individuals, mainly referred for respiratory and/or skin symptoms, were distributed in 2 groups: Group I was formed by 26 previously identified individuals reporting clinical symptoms upon contact or ingestion with tomato (3 anaphylaxis, 17 oral allergy syndrome, 6 urticaria, 2 digestive symptoms and 2 pruritus). Twenty of them had a previously positive SPT to tomato peel extract and 6 were negative.

**Figure 1:**
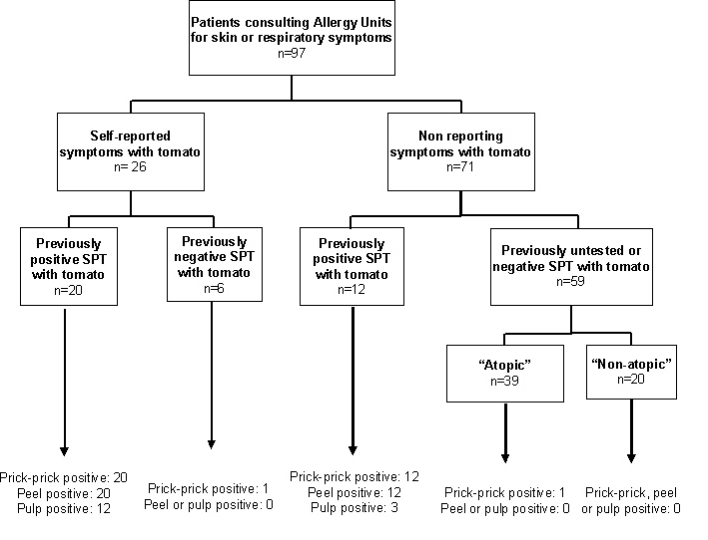
Inclusion algorithm. SPT: Skin prick test.

Group II was formed by 71 control individuals with no symptoms after tomato contact or ingestion: 12 of them were atopic and had a previously positive SPT to tomato peel extract; 59 subjects who attended to the Allergy Units during the period of the study were also included, 39 were atopic and 20 were non-atopic. The inclusion algorithm is shown in figure 1.

All patients were skin prick tested with a common battery of inhalant allergens including pollens (*Olea europaea*, grass mixture, *Artemisia vulgaris*, *Cupressus arizonica*, *Plantago lanceolata*, *Salsola kali*, *Chenopodium album*, *Parietaria judaica*, and *Platanus hybrida*); mites (*Dermatophagoides pteronissynus *and *D. farinae*); moulds (*Alternaria alternata *and *Cladosporium herbarum*) and animal danders (cat and dog epithelia). A patient was considered atopic when he/she had a positive SPT to at least one of the aeroallergens tested. All patients were also tested with a peel and pulp Canary Island tomato extract (10 mg/ml) and prick-prick tested with fresh ripe peel of Canary variety tomato. All manufactured extracts were kindly supplied by Laboratorios LETI S.L., Spain.

### Tomato extracts

The Canary Island tomato variety was selected due to its frequent consumption in Spain [10] and its high allergenicity [11]. Ripe tomatoes were purchased at a local market 2 days before preparing the extract, washed in bidistilled water and carefully peeled. 217.33 g of peel and 360.8 g of pulp were used to manufacture 2 different extracts of peel and pulp following previously described methods [12]. Briefly, after homogenization in Phosphate buffered saline/Polyvinylpolypyrrolidone (PBS/PVPP), raw materials were extracted under continuous magnetic stirring for 4 hours at 4°C, centrifuged and the supernatants collected, dialyzed, sterile filtered and freeze-dried. SPT solutions were prepared with each extract (peel and pulp) at 10 mg of freeze-dried material/ml. Freeze dried material of both extracts was reconstituted in prick solution, containing PBS 0.01 M, Glycerol 50% and Phenol 0.5%, and filtered until 0.22 μ (Millex^®^HA, Millipore, Billerica, MA, USA). Allergen extracts were characterized by SDS-PAGE and Immunoblot, using a specific pool of sera from tomato sensitized patients [9], following previously described methods [13].

### Natural Canary tomato

Canary tomatoes were purchased later on in the same local market and immediately distributed to all participants centers in December 2006. After arrival to the different centers, tomatoes were stored refrigerated at 4–8°C and used when needed during the period of study (one week).

### Prick test

SPT with tomato peel and pulp extracts and prick-prick with fresh Canary tomato were performed in all subjects simultaneously. All SPT were conducted by duplicate, in the volar surface of the forearm, in each arm in inverse order with respect to the other. SPT were performed following EAACI recommendations [14].

Prick-prick test was performed as follows: a 1 mm depth prick test lancet (Leti, Madrid, Spain) was applied to the peel of a ripe Canary tomato and the peel was torn vertically 1 cm long; afterwards, the same lancet was used to prick the patient forearm. The wheal reactions at 15 minutes were outlined with a marker and transferred to paper with transparent cello tape. Wheal sizes were measured by papulometry using the software PC Draft connected to Wacom Graphic tablet and Graphire 2 pencil (Wacon Company Ltd. Tokyo. Japan) and expressed in squared mm (± SD). Those greater or equal to 7 mm^2 ^were considered positive.

Investigators reading skin tests were not specifically informed on patient's assignation group. All the readings in the same patient were conducted by the same investigator. The exact wheal size reading (papulometry) was performed later on in all subjects by the same trained person, unaware of clinical data.

### Statistical analysis

A within subject comparison design between prick-prick test with fresh ripe Canary tomato, considered as a comparator, and manufactured freeze-dried Canary tomato peel and pulp extracts as probing solutions was performed. Concordance between results were considered the main end-point [15].

The Normality distribution of the data was calculated by Kolmogorov-Smirnov test. Non-parametric test Kruskal-Wallis One Way Analysis of Variance was used to compare the differences in the wheal sizes obtained with peel, pulp and prick-prick skin tests. Tukey's test was applied to identify the differences between pairs (p value ≤ 0.05 was considered as significant). The correlation coefficient was calculated after comparing the 3 groups in pairs. Spearman Rank correlation test was calculated in all cases. Chi-square and/or Fisher's exact test were used to compare positive tests (wheal sizes greater than 7 mm^2^) and clinical variables among groups. Student-t test was used to compare the wheal sizes between groups.

Statistical analysis was carried out using the software SigmaStat 3.5 and SigmaPlot 10.0 (Port Richmond, CA, USA).

## Results

### Population

The clinical characteristics and skin sensitizations to other allergens of the studied individuals are summarized in table 1.

**Table 1:** Characteristics of the population studied

	**Group I**	**Group II**	**P**
**n**	26	71	
**Age**	30.9 ± 12.3 (5–68)	33.9 ± 15.8 (11–75)	NS
**Sex**	3 M/23 F (11.5%/88.5%)	28 M/43 F (39.5%/61.5%)	0.013
**Atopy**	24 (92%)	51(72%)	0.053
**Sensitizations**			
**Mites**	13 (50%)	26 (37%)	NS
**Moulds**	2 (8%)	4 (6%)	NS
**Epithelia**	8 (31%)	16 (23%)	NS
**Pollens**	22 (85%)	38 (54%)	0.005
***Artemisia vulgaris***	10 (38%)	6 (8%)	0.001
***Platanus hybrida***	10 (38%)	5 (7%)	0.0005
**Grasses**	12 (46%)	17 (24%)	0.046

NS: Non-statistically significant. Non-statistically significant differences (p > 0.05) have been observed among both groups between symptoms and sensitizations to other pollens (*Olea europaea*, Chenopodiaceae, *Parietaria judaica, Cupressus arizonica *and *Plantago lanceolata*).

Group I, patients reporting symptoms with tomato, included significantly more females than group II consisting in subjects who not reported symptoms with tomato. Subjects from group I were more sensitized to pollens, especially to *A. vulgaris*, *P. hybrida *and grasses than subjects from group II. No differences in age, clinical symptoms and sensitization to non-pollen allergens were found between groups (table 1).

Subjects from group I were classified according to the prick-prick test result. The sensitization and characteristics are shown in table 2. Atopy and sensitization to pollens were significantly more frequent in patients with positive SPT to tomato. No differences in symptoms were observed between positive and negative patients, but all patients with anaphylaxis had positive skin tests.

**Table 2:** Clinical symptoms with tomato and sensitizations of the subjects from Group I (n = 26), according to the positivity of the prick-prick test with tomato

	**SPT +**	**SPT -**	**p**
**n**	21	5	
**Age**	29.4 ± 8.3 (12–41)	37.2 ± 23.1 (5–68)	NS
**Sex**	3 M/18 F (14%/86%)	0 M/5 F (0%/100%)	NS
**Atopy**	21 (100%)	3 (60%)	0.031
**Symptoms with tomato**			
**Anaphylaxis**	3	0	NS
**OAS**	14	3	NS
**Urticaria**	4	2	NS
**Digestive symptoms**	2	0	NS
**Pruritus**	1	1	NS
**Sensitizations**			
**Mites**	11 (52%)	2 (40%)	NS
**Moulds**	2 (10%)	0 (0%)	NS
**Epithelia**	8 (38%)	0 (0%)	NS
**Pollens**	20 (95%)	2 (40%)	0.014
***Olea europaea***	11 (52%)	0 (0%)	0.053
**Chenopodiaceae**	11 (52%)	0 (0%)	0.053

SPT: Skin prick test. NS: Non-statistically significant. OAS: Oral Allergy Syndrome. Non-statistically significant differences (p > 0.05) have been observed among both groups between symptoms and sensitizations to other pollens (*Artemisia vulgaris, Platanus hibrida*, grasses, *Parietaria judaica, Cupressus arizonica *and *Plantago lanceolata*).

### Characteristics of the tomato extract

At the end of the extraction process, 1.48 g of freeze-dried peel extract (yield = 0.68%) and 2.66 g of freeze-dried pulp extract (yield = 0.74%) were obtained. The peel and pulp extracts contained 84.9 and 37.5 μg of protein/mg of freeze-dried material, respectively.

**Figure 2:**
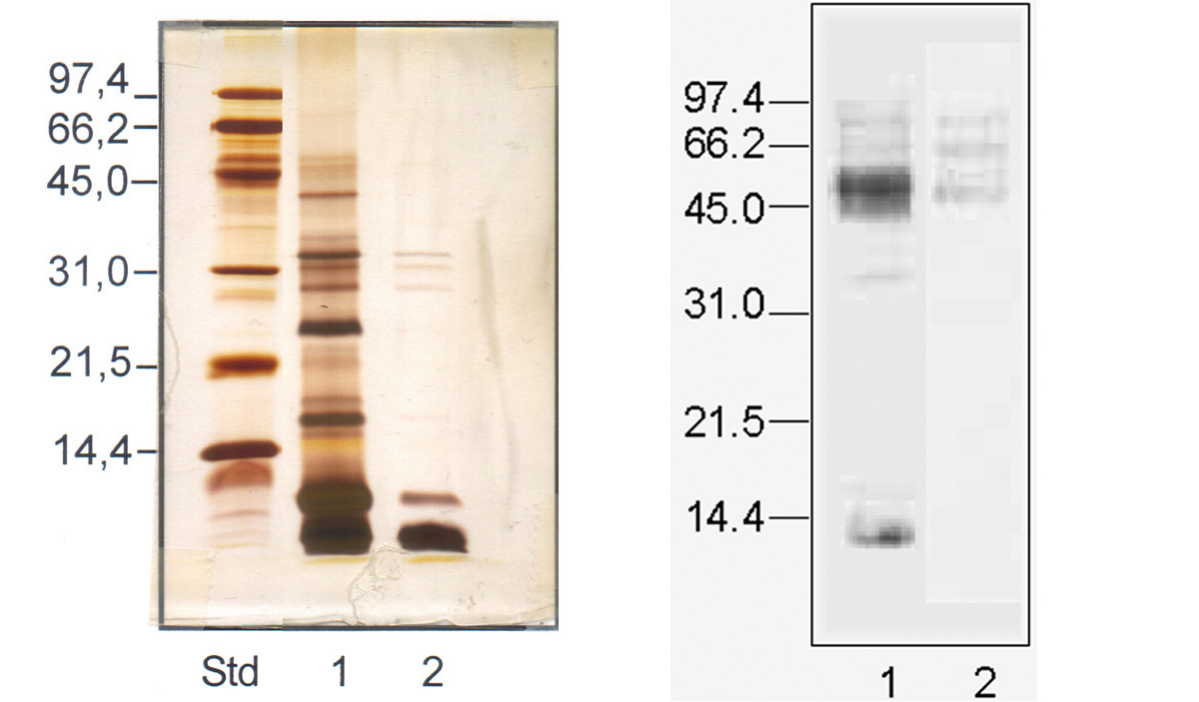
A. SDS-PAGE of the tomato extracts. Lane Std. Standard Low molecular weight (BioRad); Lane 1 Peel extract; lane 2 Pulp extract. B. Immunoblot with a specific pool of sera from tomato sensitized individuals. Solid phase: Lane 1 Peel extract; lane 2 Pulp extract.

The protein profile of the extracts revealed several bands in a molecular weight range of 9 and 50 kDa in the peel extract. The most prominent bands had molecular weights of approximately 9, 16, 25, 28, 35 and 44 kDa. The pulp extract showed 5 bands (9, 16, 28, 31 and 35 kDa). The most prominent band, in both cases, corresponds to the 9 kDa band (Figure 2A). Immunoblotting experiments showed the recognition of bands with a molecular weight between 42 and 70 kDa in peel and pulp extracts. Allergens of 32, 15 and 9 kDa were also detected in the peel extract (Figure 2B).

### Skin test positivity

Group I: Twenty-one (80.8%), of the 26 subjects, were positive to the prick-prick with ripe tomatoes. Twenty (76.9%) of them, all of the previously positive to tomato (to the tomato peel extract), were again positive to the peel extract. Twelve (46.2%) were positive to the pulp extract. None of them was only positive to the pulp extract.

Group II: The 12 (16.9%) subjects with a previous positive SPT to tomato peel were positive to both tomato peel and prick-prick. Three subjects (4.2%), all of them positive to the peel and prick-prick extract were positive to the pulp extract. One subject (1.4%) was only positive to the prick-prick tomato test.

### Wheal sizes

**Figure 3:**
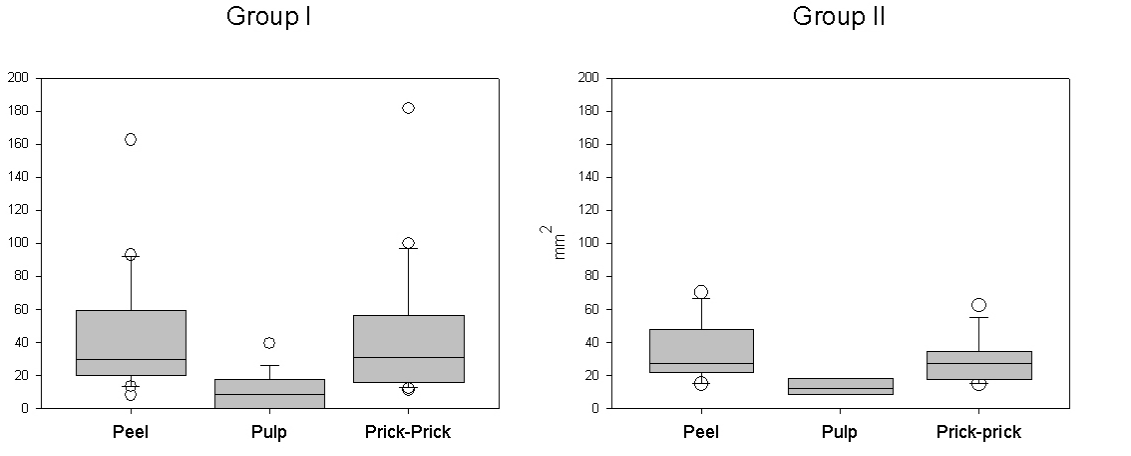
Box plot of wheal sizes induced by the different extracts in subjects from groups I and II. Horizontal lines indicate the 50th, 25–75th and 10–90th percentiles and white circles, values out of the 10–90th percentile range.

In group I, the wheal sizes induced by prick-prick among patients with positive results were 43.81 ± 40.19 mm^2^. The results for the peel extract and the pulp extract were 44.25 ± 36.68 mm^2 ^and 17.79 ± 9.39 mm^2 ^respectively (Figure 3). Kruskal-Wallis One Way Analysis of Variance on Ranks showed statistically significant differences (p < 0.05) Pairwise Multiple Comparison Procedures (Tukey Test) showed statistically significant differences between pulp and prick-prick (p < 0.01) and between pulp and peel (p < 0.01) induced wheal sizes. Non-significant differences between prick-prick and peel were obtained (p > 0.05).

**Figure 4:**
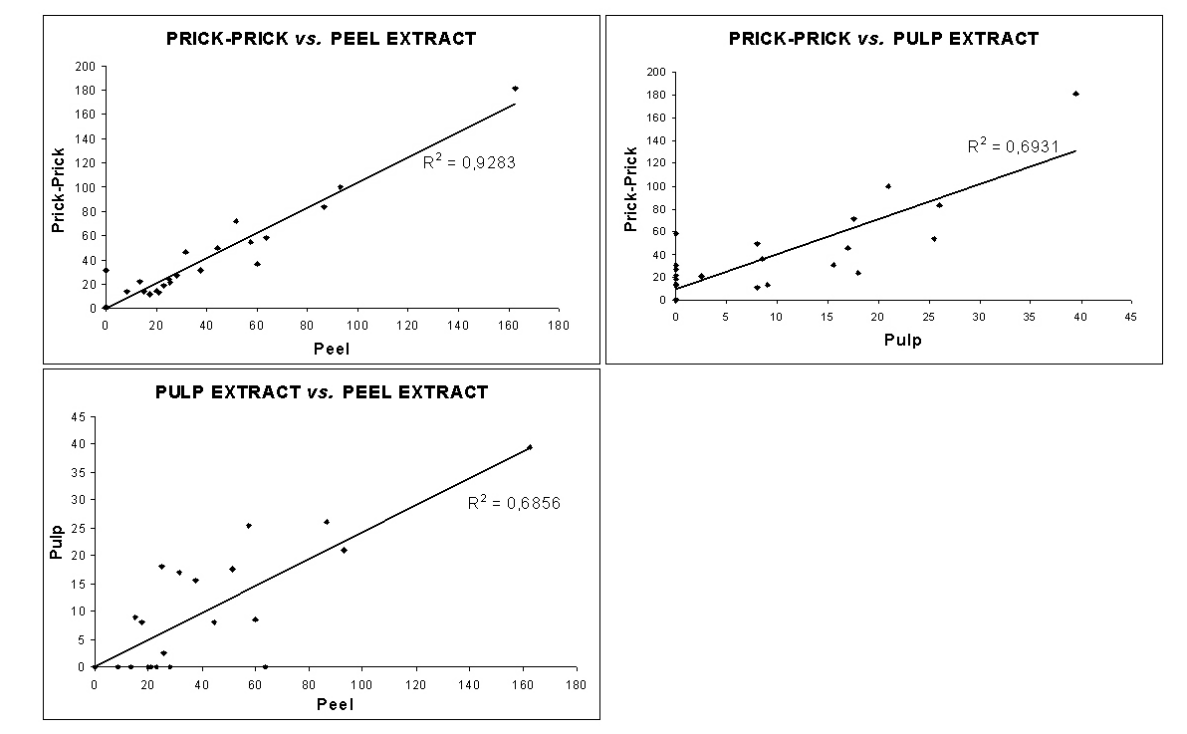
Regression curves among subjects from group I. Comparison between the different extracts. Only subjects with at least a positive skin test (wheal size ≥ 7 mm^2^) were included.

Correlation coefficients among all positive subjects were calculated. The values were prick-prick *vs*. peel: r^2 ^= 0.93; prick-prick vs. pulp: r^2 ^= 0.69 and peel vs. pulp: r^2 ^= 0.69 (Figure 4). Spearman rank correlation was significant, indicating a positive relationship between pairs (p < 0.05).

In group II, the wheal sizes induced by prick-prick among patients with positive results were 28.88 ± 13.12 mm^2 ^and 33.17 ± 17.55 mm^2 ^with tomato peel extract. Non-significant differences were observed between both groups. The wheal sizes induced by pulp extract were 13.33 ± 4.80 mm^2^. Significant differences were obtained between pulp and peel and prick-prick (p < 0.05).

There were not statistically significant differences in wheal sizes between individuals with positive SPT from group I and group II.

## Discussion

The use of commercial extracts for skin testing in the diagnosis of sensitivity or allergy to foods, especially fruits and vegetables, is frequently considered of limited value due to the use of non-standardized extracts. In addition, the use of natural fresh foods may have disadvantages due to the variability of the source material, the low reproducibility of the test and the poor availability of the material.

In the present study, we found a good correlation between peel tomato extract and pick-prick tests, showing nearly identical mean values. However, skin testing with pulp extract showed a poorer correlation compared with prick-prick test.

Canary tomato variety was selected due to its higher protein content compared to other tomato varieties [11] and to its high sensitivity in SPT, inducing the largest wheals when compared with other varieties [16]. There are no studies comparing manufactured prick tests and prick-prick tests with fresh tomatoes, but our results replicate those found by several authors with different fruits (apple and peach for example) [3,4,7]. Several studies have shown that available commercial extracts may loose relevant allergens during the manufacturing process.) [3,4], specially when several important parameters are not well controlled such as the extractant solution, the temperature or the pH. Therefore, while standardized food extracts are still in most cases unavailable, new manufactured extracts should be assessed for the presence of all relevant allergens and tested against fresh foods, the best approximation to a gold standard in this field.

In our study, we have found a good correlation between peel tomato extract and prick-prick. Only two subjects had positive prick-prick to tomato and negative to peel tomato extract. Both of them were atopic patients, polysensitized to pollens and others inhalants; one of them, reporting oral allergy syndrome (OAS) with tomato. It has been demonstrated [8] in birch sensitized patients, that some relevant allergens could not be enough represented in the extracts. This could be the case of these two subjects.

The selection of patients could be biased by the need to recruit patients in the shortest period in order to provide uniform experimental conditions (same batch of tomatoes...) in all subjects. Thus, individuals from group I were selected among individuals that previously reported symptoms with tomato and had been previously tested with tomato extracts. In addition, a group of control subjects was included because of their previously positive SPT to tomato. The inclusion of these asymptomatic sensitized patients was important to assess the characteristics of the skin sensitizations in this population. Even if the number of subjects and the selection of individuals make difficult to compare results between positive individuals from groups I and II (asymptomatic), the mean wheal sizes in both groups have been similar. This fact could be related either to the absence of significant differences between the two groups or to the lack of enough power to detect them. The extracts used for detecting these asymptomatic but "previously sensitized" subjects and the probing extracts used in the study have been the same, so, the subset of asymptomatic subjects only positive to prick-prick could be under-represented. In our study, prick-prick only detected 1.5% more of the control group subjects and 4% of the patients reporting symptoms with tomato than the peel extract. As tomato allergy has not been confirmed with challenge tests, we cannot use self-reported symptoms as a gold standard and the prick-prick method was chosen as comparator to assess sensitization to tomato. The number of negative patients reporting symptoms with tomato is low in this study, but they have some differences with subjects positive to tomato extracts (less atopic and less sensitized to pollens) that could suggest the possibility of non-allergic symptoms.

The high correlation and concordance found between prick-prick and the peel extract suggests that, at least in our population, the manufactured peel extract can be considered a useful tool for detection of tomato sensitization. While skin testing with fresh foods should not been completely abandoned, better extracts for diagnosis may render this sometimes inconvenient procedure unnecessary in most cases, reserving it only for cases of discordance between self-reported symptoms and negative SPT with previously manufactured extracts: According to our results, the use of commercial skin prick test detected 77% of subjects who reported symptoms; Performing additional skin testing with fresh tomatoes in the remaining 23% would only slightly increase the number of detected subjects up to 81%. Until standardized food extracts, with known biological activity and/or major allergen content [3] are available, carefully manufactured food extracts, much more available and reproducible than fresh food, warrant their use at any moment and should improve the diagnosis of food allergy and food sensitization.

## Conclusion

We have shown that carefully manufactured tomato peel and pulp extracts are useful and reliable in the diagnosis of tomato sensitization. Wheal sizes induced by peel extracts were highly correlated with those obtained with prick-prick testing. Therefore we conclude that the peel extract is a good alternative for *in vivo *diagnosis of tomato sensitization.

## Competing interests

The author(s) declare that they have no competing interests.

## Authors' contributions

Ferrer A, Huertas AJ, Larramendi CH, Fernández-Caldas E and Carnés J have designed the study, based on previous discussions between all authors. Ferrer A, Huertas AJ, Larramendi CH, García-Abujeta JL, Bartra J, Lavín JR, Andreu C and Pagán JA have equally contributed in the selection and inclusion of patients. Fernández-Caldas E and Carnés J have performed all the laboratory procedures. Ferrer A, Huertas AJ, Larramendi CH and Carnés J have extensively worked on the draft versions of the paper. All authors have revised the final version.

## References

[B1] Bousquet J, Anto JM, Bachert C, Bousquet PJ, Colombo P, Crameri R, Daeron M, Fokkens W, Leynaert B, Lahoz C, Maurer M, Passalacqua G, Valenta R, van Hage M, Van Ree R (2006). Factors responsible for differences between asymptomatic subjects and patients presenting an IgE sensitization to allergens. A GA2LEN project. Allergy.

[B2] Asero R, Ballmer-Weber BK, Beyer K, Conti A, Dubakiene R, Fernandez-Rivas M, Hoffmann-Sommergruber K, Lidholm J, Mustakov T, Oude Elberink JN, Pumphrey RS, Stahl Skov P, van Ree R, Vlieg-Boerstra BJ, Hiller R, Hourihane JO, Kowalski M, Papadopoulos NG, Wal JM, Mills EN, Vieths S (2007). IgE-Mediated food allergy diagnosis: Current status and new perspectives. Mol Nutr Food Res.

[B3] Cuesta-Herranz J, Lázaro M, Martínez A, Álvarez-Cuesta E, Figueredo E, Martínez J, Cuesta C, de las Heras M (1998). A method for quantitation of food biologic activity: results with peach allergen extracts. J Allergy Clin Immunol.

[B4] Ferrer A, Carnés J, Gallego MT, Andreu C, Fernández-Caldas E (2005). Characterization and improvement of apple extracts for the diagnosisof apple IgE-mediated allergy. Ann Allergy Asthma Immunol.

[B5] Ortolani C, Ispano M, Pastorello EA, Ansaloni R, Magri GC (1989). Comparison of results of skin prick tests (with fresh foods and commercial food extracts) and RAST in 100 patients with oral allergy syndrome. J Allergy Clin Immunol.

[B6] Larramendi CH (2003). Food allergy diagnosis: Are there any missing factors?. A theoretical approach. Med Hypotheses.

[B7] Asero R, Mistrello G, Roncarolo D, Antoniotti P, Cislaghi C, Falagiani P (1999). A new apple extract. Allergy.

[B8] Asero R, Mistrello G, Roncarolo D, Casarini M, Falagiani P (2001). Allergy to nonspecific lipid transfer proteins in Rosaceae: a comparative study of different in vivo diagnostic methods. Ann Allergy Asthma Immunol.

[B9] Larramendi CH, Ferrer A, Huertas AJ, García-Abujeta JL, Andreu C, Tella R, Cerdà MT, Bartra J, Lavín JR, Pagán JA, López-Matas MA, Fernández-Caldas E, Carnés J (2008). Sensitization to tomato peel and pulp extracts in the Mediterranean Coast of Spain. Prevalence and co-sensitization with aeroallergens. Clin Exp Allergy.

[B10] Illescas JL, Bacho O (2005). Análisis de las principales variedades de hortalizas y patatas. Evolución y tendencias en los mercados de frutas y hortalizas (II): Tomate. Distribución y Consumo.

[B11] Carnés J, López-Matas M, Ferrer A, Larramendi C, Huertas J, Casanovas M, Fernández-Caldas E (2006). Immunochemical Characterization of Tomato Peel and Pulp Extracts [abstract]. J Allergy Clin Immunol.

[B12] Carnés J, Fernández-Caldas E, Gallego MT, Ferrer A, Cuesta-Herranz J (2002). Pru p 3 (LTP) content in peach extracts. Allergy.

[B13] Laemmli UK (1970). Cleavage of structural proteins during the assembly of the head of bacteriophage T4. Nature.

[B14] Dreborg S, Sub-Committee on Skin Tests of the European Academy of Allergology and Clinical Immunology (1989). 2. Methods for skin testing. Allergy.

[B15] The European Agency for the Evaluation of Medicinal Products (EMEA) (2007). Points to Consider on the Evaluation of Diagnostic Agents.

[B16] Ferrer A, Hernando de Larramendi C, Pagán J, Huertas J, Bartra J, Andreu C (2006). Análisis *in vivo *de la alergenicidad de 7 variedades de tomate [abstract]. J Investig Allergol Clin Immunol.

